# Comparison of analogue and digital mammographic appearances of screen-detected invasive breast cancers

**DOI:** 10.1186/bcr2956

**Published:** 2011-11-04

**Authors:** TW Jones, G Bansal, H Farmer, B Orr, H Russell, L Hobson, D Godden, I Lyburn

**Affiliations:** 1 Gloucestershire Breast Screening Service, Cheltenham, UK

## Introduction

Our UK Breast Screening Service changed overnight wholesale from analogue to digital mammography on 5 October 2009. This has meant that we have two unmixed cohorts to directly compare. We wish to evaluate whether any digital mammography has changed our cancer detection, and whether any features of these cancers have altered.

## Methods

A NBSS database search for all screen detected cancers in the periods 1 year prior to the digital changeover; and 1 year after. This period was sufficiently historical to expect all cancer diagnosis episodes to have closed by the time of study. The screening packets for all these patients were pulled, and films and pathology analysed by the researchers. The information was directly entered into an anonymised spreadsheet.

## Results

For 2008 to 2009 analogue: 24,876 women invited, 20,557 screened, 944 recalled for assessment, 155 diagnosed with cancer; mean age 59.7. For 2009 to 2010 digital: 32,143 women invited, 25,088 screened, 1,230 recalled for assessment, 221 diagnosed with cancer; mean age 61.0.

## Conclusion

Screening uptake figures were high in this unit (78% and 82% for each group). Cancer detection rates were significantly increased in the age 50 to 64 group (analogue 4.6 and digital 6.2/1,000 women screened). Mean cancer sizes were smaller on digital mammography but this also corresponded with smaller mean pathological sizes. Adjusting for this, the digital system still identified smaller cancers than the analogue system. Overall, cancer detection seems improved by our change to digital mammography.

